# Mild traumatic brain injury induces memory deficits with alteration of gene expression profile

**DOI:** 10.1038/s41598-017-11458-9

**Published:** 2017-09-07

**Authors:** Yawen Luo, Haiyan Zou, Yili Wu, Fang Cai, Si Zhang, Weihong Song

**Affiliations:** Townsend Family Laboratories, Department of Psychiatry, The University of British Columbia, 2255 Wesbrook Mall, Vancouver, BC, V6T 1Z3 Canada

## Abstract

Repeated mild traumatic brain injury (rmTBI), the most common type of traumatic brain injuries, can result in neurological dysfunction and cognitive deficits. However, the molecular mechanisms and the long-term consequence of rmTBI remain elusive. In this study, we developed a modified rmTBI mouse model and found that rmTBI-induced transient neurological deficits and persistent impairments of spatial memory function. Furthermore, rmTBI mice had long-lasting detrimental effect on cognitive function, exhibiting memory deficits even 12 weeks after rmTBI. Microarray analysis of whole genome gene expression showed that rmTBI significantly altered the expression level of 87 genes which are involved in apoptosis, stress response, metabolism, and synaptic plasticity. The results indicate the potential mechanism underlying rmTBI-induced acute neurological deficits and its chronic effect on memory impairments. This study suggests that long-term monitoring and interventions for rmTBI individuals are essential for memory function recovery and reducing the risk of developing neurodegenerative diseases.

## Introduction

Traumatic brain injury (TBI), a brain damage caused by an external mechanic force, is a serious issue to public health. It is the leading cause of death and disability for people under 45. Based on severity, TBI is classified into mild, moderate and severe TBI. An estimated 57 million people have experienced TBI worldwide. The majority of TBI cases, accounting for more than 80%, are mild. Although mild TBI (mTBI) is not the primary cause of mortality, it does contribute to acute neurological deficits and chronic persistent neurological symptoms and cognitive deficits, including confusion, impaired consciousness and memory deficits. Growing evidence suggests that repeated mTBI (rmTBI) may have a great cumulative effect on brain functions, leading to neuropsychological disabilities, and significantly increases the risk of developing neurodegenerative disorders, such as Alzheimer’s disease and Parkinson’s disease^1–3^.

rmTBI, often occurring in sports, e.g. boxing, soccer, hockey and American football, mainly affects adolescents and young adults^4^. In addition, victims of child abuse and spousal abuse also contribute to rmTBI^5, 6^. Although long-term impaired brain functions are believed to be associated with rmTBI, even when incidents are separated in time by months or years, the exact effect of rmTBI on brain functions and the underlying mechanism remain elusive^7^. Thus, it is imperative to determine the long-term impairment-induced by rmTBI, define the underlying molecular mechanisms, and develop effective intervention approaches or treatments.

Although the information of cognitive assessments and brain imaging from mTBI patients is available, mTBI studies in human are challenged by the following difficulties. First, mTBI patients may not be admitted or seen by a specialist. Second, the difference of brain imaging may not be detected in patients with clinical signs and symptoms. In addition, long-term effects of mTBI have not be well documented, such as a mild memory decline. Moreover, the molecular studies of mTBI in patients are limited by the lack of human samples. Thus, animal models of mTBI are valuable tools to investigate cellular and molecular mechanisms of mTBI and to monitor long-term effects of mTBI on cognitive functions. Among the species of TBI models, rodent models are primarily being used. Four typical TBI animal models include fluid percussion injury model, blast injury model, weight-drop model and controlled cortical impact model. Compared with others, weight-drop mouse model is a close-head and non-invasive model. Weight-drop model mimics mTBI in human, including two key factors, high velocity and rapid acceleration^8–15^.

Although increased animal model-based studies of mTBI have been reported, most of them focused on short-term effect, in the range of hours to 10 days post-mTBI^16–18^. However, changes at early time points may not fully represent the long-term outcome. A few studies showed that long-term changes can last for more than 1 year after mTBI in animals and patients^19–22^. More importantly, TBI, including rmTBI, even occurring at young age, significantly increases the risk of neurodegenerative diseases^1–3^.

Most studies on the cellular and molecular processes by TBI focused on examining the expression of individual genes involved in the impairment and recovery of brain functions^23, 24^. Gene expression microarray is a powerful approach to determine the alteration of whole genome gene expression, which not only indicates the change of each individual gene but also directly indicates the correlation among each gene and each molecular pathway. Accordingly, the affected biological functions can be predicted and validated. Recently, several whole genome gene expression studies have been performed to define the molecular changes of TBI in animal models and *in vitro* models^25^. However, although rmTBI is the most common form of TBI, the alteration of gene expression profiles and molecular pathways in rmTBI has not been investigated.

In the present study, we first examined both short-term and long-term effects of rmTBI on memory functions by employing a modified weight-drop model of rmTBI on mice. We found that significant memory deficits were detected at 2, 8 and 12 weeks after rmTBI. Microarray analysis suggested that rmTBI significantly altered the expression of 87 genes involved in apoptosis, metabolism, transcription, protein trafficking, stress response and synaptic plasticity.

## Results

### Acute neurological impairment is not exaggerated by rmTBI

**Figure 1:**
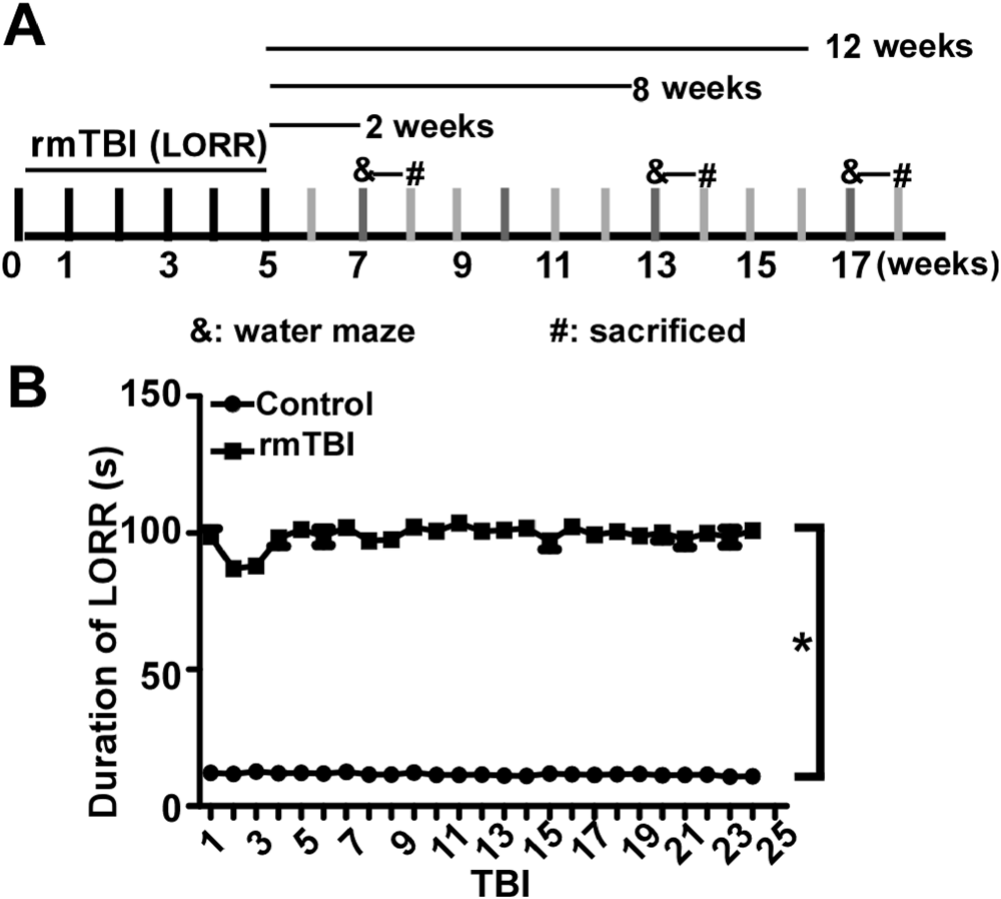
rmTBI does not exaggerate the increase of the duration of LORR. (**A)** Schematic diagram of the experiments. All the rmTBI mice received the concussion-like head injury once a day from Monday to Friday for 5 uninterrupted weeks, while the control mice received sham treatment. After the 25 repeated head injuries, Morris water maze (MWM) was performed at the 2^nd^, 8^th^ and 12^th^ week after the last injury, respectively. & represents the time point of MWM and **#** represents the time point of mice being sacrificed. (**B**) The duration of LORR was measured after each mTBI (rmTBI) and sham treatment (Control). The number represents the duration of LORR after single mTBI. The values are expressed as mean ± SEM, N = 17/group. *p < 0.05 by two-way ANOVA.

To examine the acute neurological responses, the duration of the loss of righting reflex (LORR) in rmTBI and control mice were recorded. rmTBI mice received the concussive-like head injury once a day for 5 uninterrupted weeks, while control mice were treated following the same procedure except for concussive-like head injury (Fig. 1A). One mouse displaying forelimb paralysis was excluded. The duration of LORR was recorded after each injury. After single mTBI, the average duration was 98.71.70 ± 4.87 seconds in mTBI mice, while the control mice had an average duration of 12.23 ± 1.6 2 seconds, p < 0.05 after the 1^st^ TBI (Fig. 1B). The duration of LORR was not altered by the number of injuries, 100.94 ± 4.51 seconds after 25^th^ mTBI compared with 98.71.70 ± 4.87 seconds on day 1, p > 0.05 (Fig. 1B). However, both single mTBI and rmTBI significantly increased the duration of LORR compared with the sham treatment, p < 0.05 (Fig. 1B). This result demonstrates that mTBI significantly increased the duration of LORR compared with the sham treatment. However, rmTBI did not exaggerate the increase of the duration of LORR (Fig. 1B). More importantly, no death and skull fracture occurred in rmTBI mice.

### rmTBI induces persistent memory deficits

**Figure 2:**
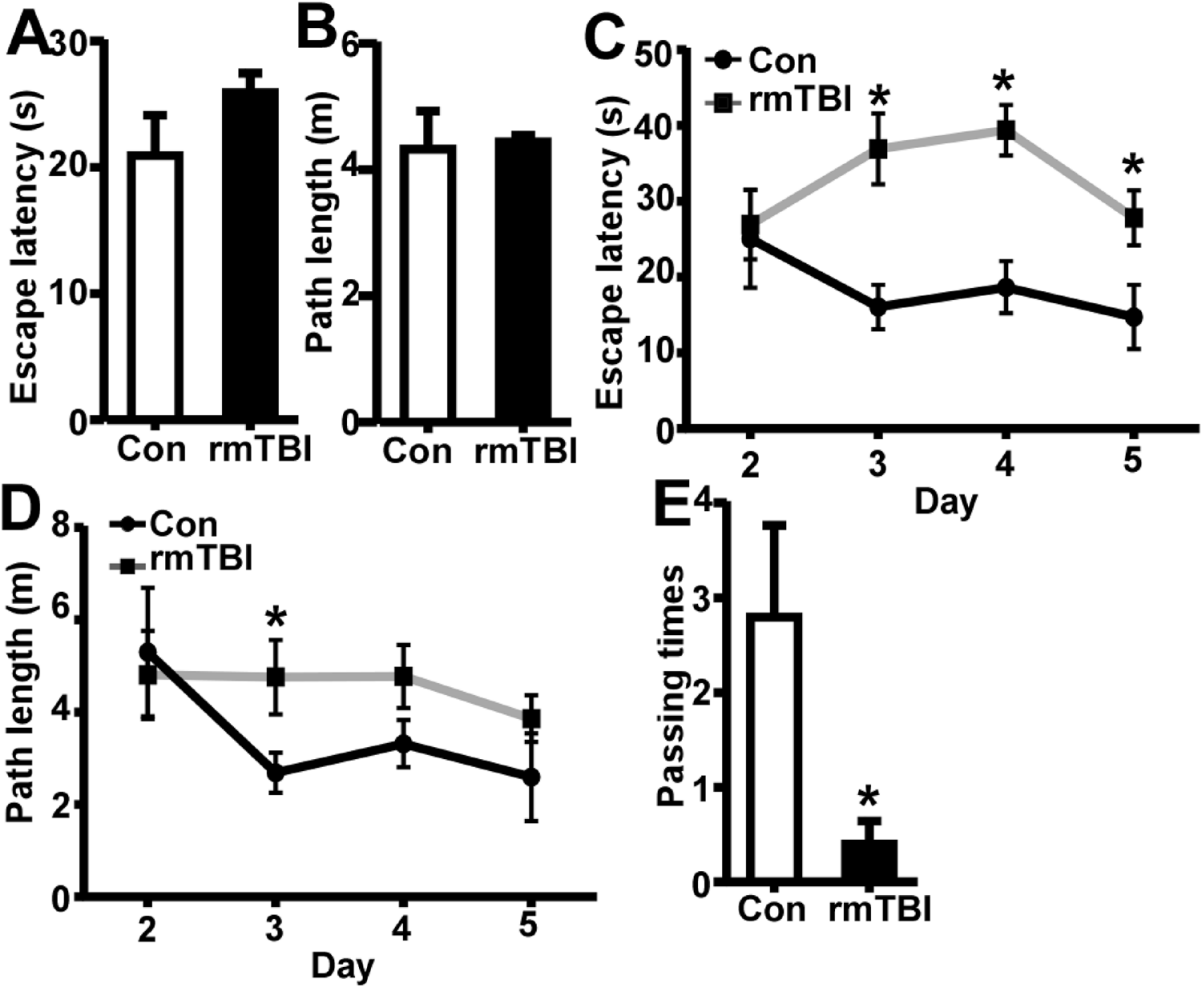
Memory deficits at 2 weeks after the last injury of rmTBI. A Morris water maze test consists of 1 day of visible platform tests, 4 days of hidden platform tests and 1 probe test 24 hours after the last hidden platform test. In the visible platform tests, rmTBI and control mice had similar latency (**A**) and swimming distance (**B**) to escape onto the platform. The values were expressed as mean ± SEM, N = 5/group, p > 0.05 by Student’s t-test. In the hidden platform test, mice were trained with 5 trials per day for 4 days. rmTBI mice had longer latency (**C**) and swimming distance (**D**) on the 3^rd^, 4^th^ and 5^th^ day. The values were expressed as mean ± SEM, N = 5/group, *p < 0.05 by ANOVA. (**E**) In the probe trial, the number of times the rmTBI mice traveled into the platform zone was significantly less than that of the control mice. The values were expressed as mean ± SEM, N = 5/group, *p < 0.05 by Student’s t-test.

Since memory impairment is among the top complaints of patients with rmTBI, next we examined whether rrmTBI lead to memory deficits in this modified rmTBI mouse model. Two weeks after the last injury, the effect of rmTBI on spatial memory was determined by Morris water maze. To rule out the possibility that the performance of rmTBI mice in water maze was affected by muscular strength and balance ability, wire hanging test was utilized to examine the muscular strength and balance ability before the water maze test. The mice in both control group and rmTBI group hung on the wire for at least 60 seconds, indicating that there was no motor dysfunction in rmTBI mice. In the visible platform test of Morris water maze, rmTBI and control mice had similar escape latency (20.93 ± 3.20 and 25.92 ± 1.54 s, P > 0.05 (Fig. 2A) and path length (4.33 ± 0.61 and 4.44 ± 0.11 m; P > 0.05) (Fig. 2B), indicating that rmTBI did not affect mouse mobility or vision. In the hidden platform-swimming tests, the escape latency was significantly longer in rmTBI mice than that in control mice from day 3 to day 5, 36.93 ± 4.66 vs. 16.04 ± 2.93 on day 3, 39.39 ± 3.35 vs. 18.65 ± 3.43 on day 4 and 27.82 ± 3.62 vs.14.73 ± 4.27 son day 5 (P < 0.05) (Fig. 2C). The rmTBI mice swam significantly longer distances to reach the platform (4.76 ± 0.80, 4.77 ± 0.68 and 3.86 ± 0.50 m) compared with the control mice (2.69 ± 0.43, 3.32 ± 0.51 and 2.59 ± 0.95 m) on the 3^rd^, 4^th^ and 5^th^ day (P < 0.05) (Fig. 2D). In the probe trial on the last day of testing, the number of times rmTBI mice traveled into the platform zone, where the hidden platform was previously placed, was significant less compared with that in control mice, 0.40 ± 0.24 vs. 2.80 ± 0.97 (P < 0.05) (Fig. 2E). These data demonstrated that spatial memory was significantly impaired in rmTBI mice compared with that in control mice at two weeks after the last injury.

**Figure 3:**
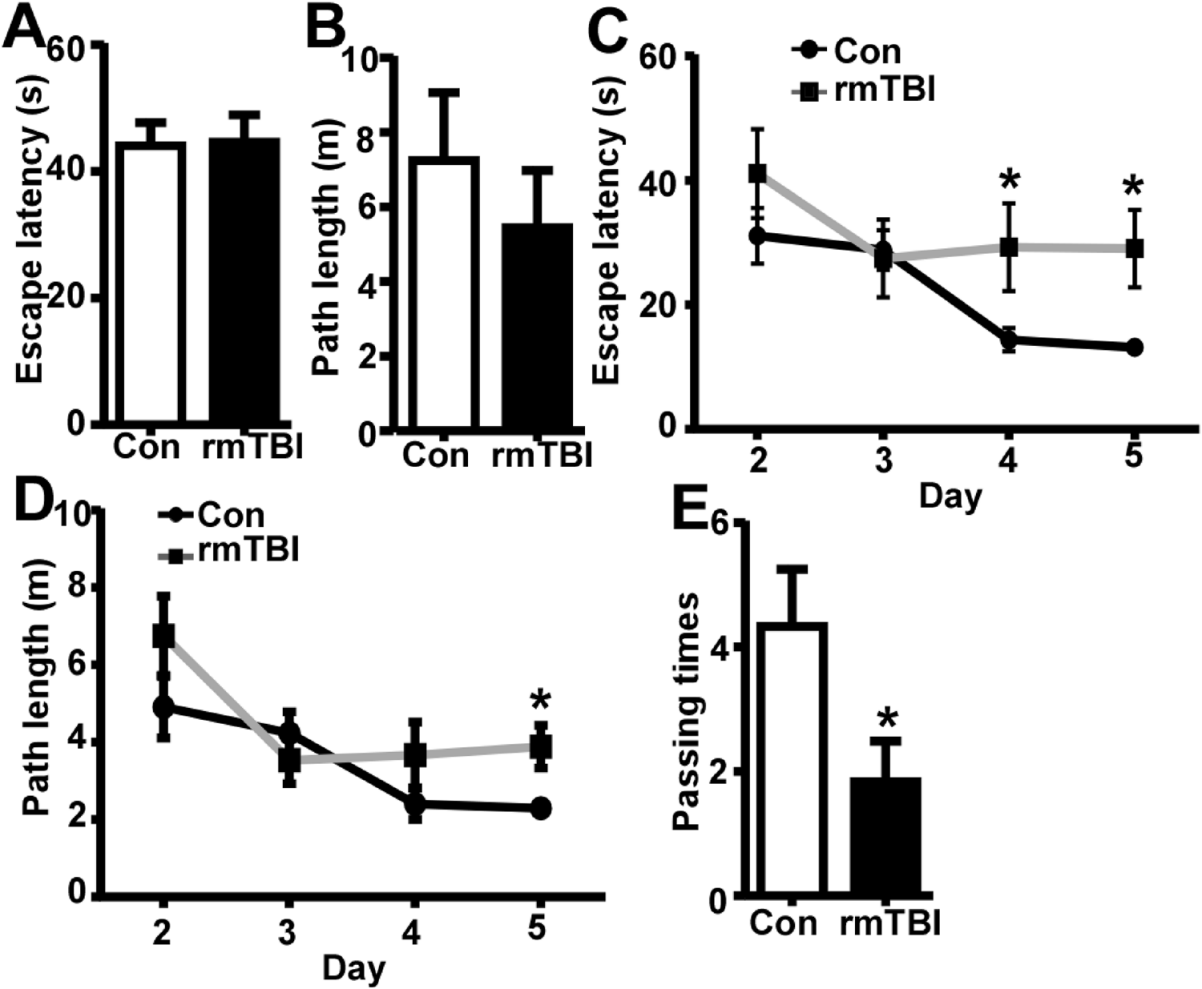
Memory deficits at 8 weeks after the last injury of rmTBI. In the visible platform tests, rmTBI and control mice had similar latency (**A**) and swimming distance (**B**) to escape onto the platform. The values were expressed as mean ± SEM, N = 5/group, p > 0.05 by Student’s t-test. In the hidden platform test, rmTBI mice had longer latency (**C**) and swimming distance (**D**) on the 5^th^day. The values were expressed as mean ± SEM, N = 6/group, *p < 0.05 by ANOVA. (**E**) In the probe trial, rmTBI mice traveled into the platform zone fewer times than control mice. The values were expressed as mean ± SEM, N = 6/group, *p < 0.05 by Student’s t-test.

Eight weeks after injury, the effect of rmTBI on spatial memory was also examined by Morris water maze. In the visible platform test of Morris water maze, rmTBI and control mice had similar escape latency (44.62 ± 4.32 and 44.1 ± 3.63 s, P > 0.05) (Fig. 3A) and path length (5.45 ± 1.53 and 7.25 ± 1.82 m, P > 0.05)(Fig. 3B), indicating that rmTBI did not affect mouse mobility or vision at this point. In the hidden platform-swimming tests, the escape latency was significantly longer in rmTBI mice than that in control mice on day 5, 29.07 ± 6.23 vs.13.2 ± 0.87 s (P < 0.05)(Fig. 3C). The rmTBI mice swam significantly longer distances to reach the platform compared with the control mice on the 5^th^ day, 3.89 ± 0.55 vs. 2.29 ± 0.16 m (P < 0.05) (Fig. 3D). In the probe trial, the number of times rmTBI mice traveled into the platform zonewas significant less than that in control mice, 1.83 ± 0.65 vs. 4.33 ± 0.92 (P < 0.05) (Fig. 3E). These data demonstrated that spatial memory was significantly impaired in the rmTBI mice at 8 weeks after the last injury.

**Figure 4:**
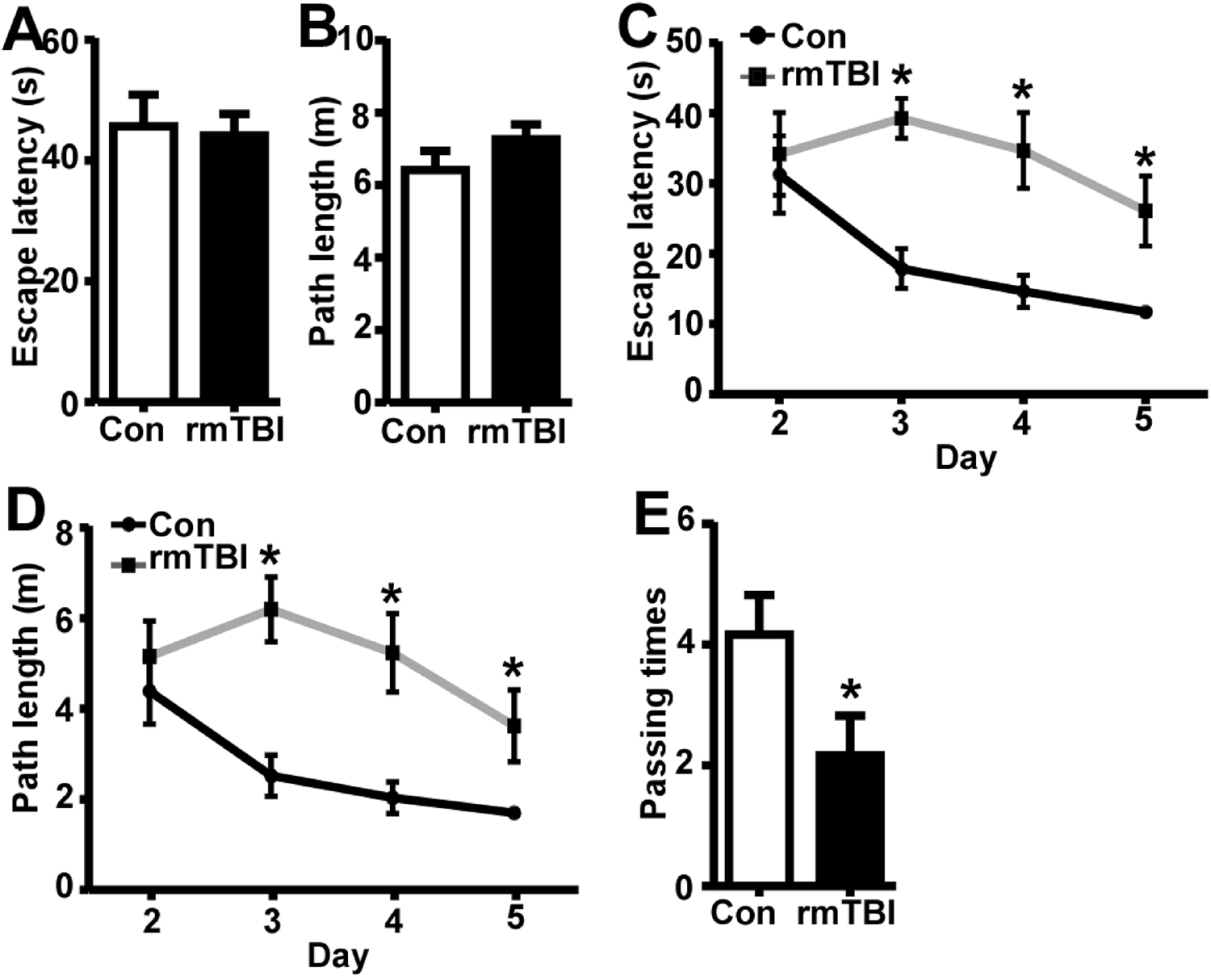
Memory deficits at 12 weeks after the last injury of rmTBI. In the visible platform tests, rmTBI and control mice had similar latency (**A**) and swimming distance (**B**) to escape onto the platform. The values were expressed as mean ± SEM, N = 5/group, p > 0.05 by Student’s t-test. In the hidden platform test, rmTBI mice had longer latency (**C**) and swimming distance (**D**) on the 3^rd^, 4^th^ and 5^th^ day. The values were expressed as mean ± SEM, N = 6/group, *p < 0.05 by ANOVA. (**E)** In the probe trial, rmTBI mice traveled into the platform zone less times than control mice. The values were expressed as mean ± SEM, N = 6/group, *p < 0.05 by Student’s t-test.

To further investigate the chronic effect of rmTBI on memory functions, Morris water maze was performed at twelve weeks after the last injury. In the visible platform test of Morris water maze, rmTBI and control mice had similar escape latency (44.13 ± 3.61 and 45.67 ± 5.24 s, P > 0.05) (Fig. 4A) and path length (7.27 ± 0.40 and 6.42 ± 0.53 m, P > 0.05) (Fig. 4B). In the hidden platform-swimming tests, the escape latency was significantly longer in rmTBI mice than that in control mice from day 3 to day 5, 39.27 ± 2.83 vs. 17.9 ± 2.83 s on the 3^rd^ day, 34.7 ± 5.40 vs. 14.63 ± 2.29 s on the 4^th^ day and 26.1 ± 4.9811.73 ± 0.61 son the 5^th^ day (P < 0.05) (Fig. 4C). The rmTBI mice swam significantly longer distances to reach the platform, 6.20 ± 0.71 vs. 2.52 ± 0.45, 5.24 ± 0.86 vs. 2.04 ± 0.34 and 3.62 ± 0.79 vs. 1.70 ± 0.07 mon the 3^rd^, 4^th^ and 5^th^ day (P < 0.05) (Fig. 4D). In the probe trial, the number of times rmTBI mice traveled into the platform zone was significant less compared with that in control mice, 2.17 ± 0.65 vs. 4.17 ± 0.65 (P < 0.05) (Fig. 4E). These data demonstrated that spatial memory was persistently impaired in rmTBI mice even 12 weeks after the last injury.

### Gene expression profiling in rmTBI mice and functional classification

**Figure 5:**
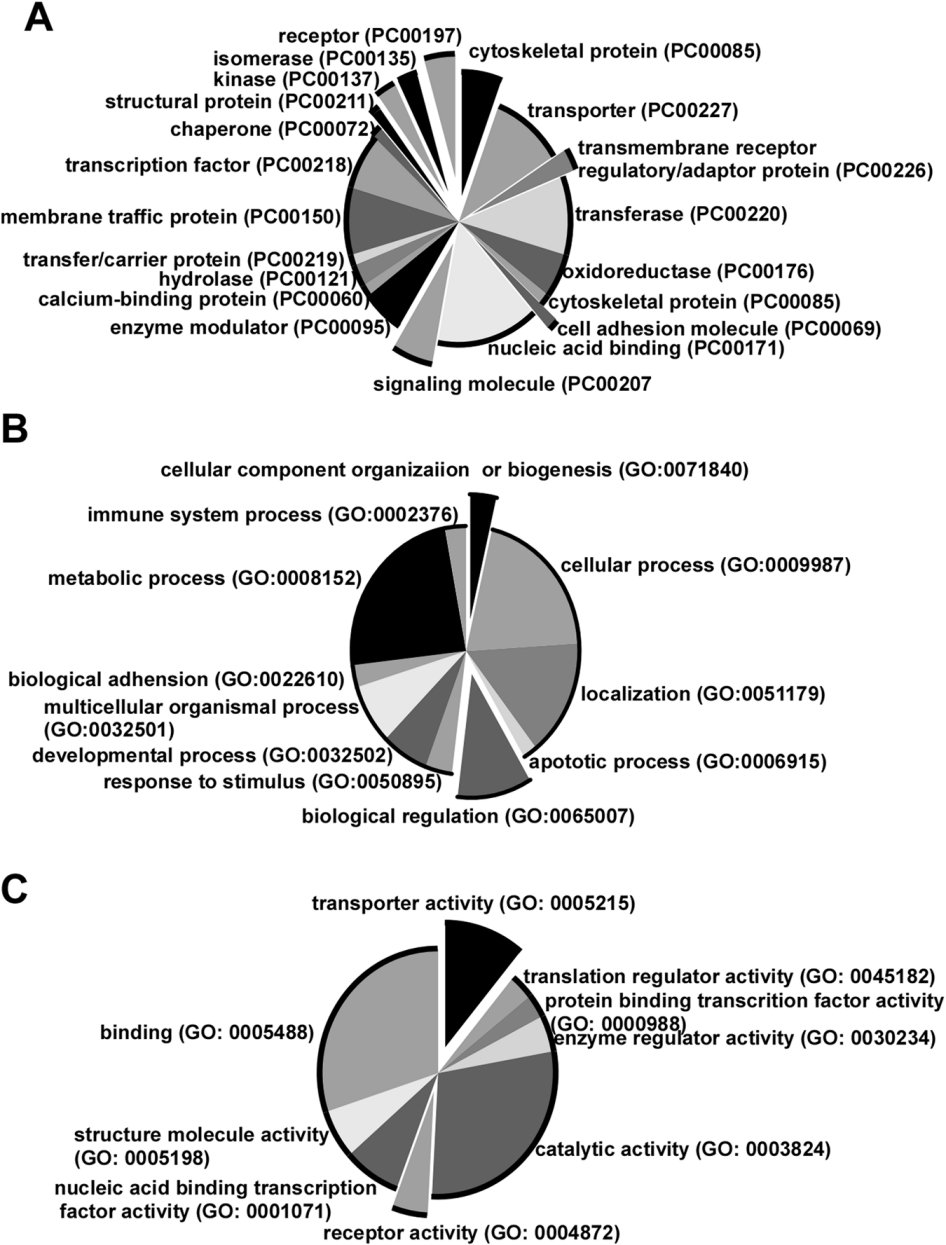
Functional classification of differentially expressed genes in the hippocampi. 3 weeks after rmTBI, the whole genome gene expression of the hippocampi of rmTBI and control mice was profiled with Illumina’s Sentrix® Mouse-6 Expression Bead chips. 87 differentially expressed genes were extracted with Beadstudio software and subjected to Panther 9.0 software for functional analysis. (**A)** Protein classification of the differentially regulated genes in rmTBI mice. (**B)** The differentially expressed genes were classified in to 11 categories of biological process according to GO terms. (**C)** The differentially expressed genes were classified into 9 molecular functions according to GO terms.

To elucidate the molecular mechanism of rmTBI-induced memory deficits, gene expression profiling was performed on hippocampus of rmTBI mice and control mice after the behavioral tests. 2 weeks after the last injury, Morris water maze was performed in rmTBI mice and control mice. Microarray experiments were performed one day after the Morris water maze tests. 87 genes were identified to be differentially expressed at cut-off 1.2 folds (p < 0.05) in TBI mice (Supplementary Table 1). Among them, 43 genes were up-regulated and 44 genes were down-regulated. The encoding proteins were classified into nucleic acid binding, transcription factor, transporter, receptor and membrane traffic protein etc. categories (Fig. 5A), suggesting that they play an important role in biological processes and molecular functions. Moreover, functional classification analysis showed that the differentially expressed genes belonged to apoptotic process, response to stimulus, metabolic process, developmental process and biological regulation etc. biological processes (Fig. 5B). Furthermore, they represented a diverse spectrum of molecular functions, including translation regulator activity, transcription factor activity, catalytic activity and enzyme regulator activity etc. (Fig. 5C). Although no signaling pathway was significantly affected, the differentially regulated genes did involve in synaptic vesicle trafficking, ionotropic glutamate receptor pathway, opioid prodynorphin pathway, and Huntington disease and Parkinson disease pathways. These data indicated that rmTBI significantly altered the expression of many key molecules which may have a broad effect on brain functions and diseases pathogenesis, including memory function and neurodegenerative disorders.

## Discussion

Although the information of cognitive assessments and brain imaging from mTBI patients is available, mTBI studies in human are challenged by many difficulties. Animal models of mTBI are useful to investigate the cellular and molecular mechanisms of mTBI and monitor long-term effects of mTBI on cognitive functions, providing better understanding of the neurobiological and behavioral outcomes of mTBI, and helping the development of effective therapeutic approaches. Among the species of TBI models, rodent models are primarily being used. The classical weight-drop model, Marmarou model, causes a high rate of skull fracture and mouse death^26^. In this study, we developed a modified weight-drop model with a 20 g weight and a 25 cm height according to the previous study^18^, Notably, no skull fracture and mouse death occurred in all TBI mice. In addition, the motor function was not affected in this mTBI model. It indicates that this model is better than the classical mTBI model. Although a few of modified weight-drop models of rmTBI with different injury times and intervals have been reported, the injury times are between 2 to 5, which are still not sufficient to represent the situation in human individuals who often experienced far more times of injuries, such as sports-related rmTBI in boxing, soccer, hockey and football etc. Therefore, we developed a new rmTBI model in which the mice were injured once a day for 25 uninterrupted days. More importantly, this novel model with milder injury and extended injury days did not impair muscular strength, balance ability and vision mice according to their performance of wire hang test and the first day test of Morris water maze. Using this new model, we found that a single mTBI significantly induced acute neurological dysfunction by increasing the duration of LORR. However, the uninterrupted injuries, up to 25 times, did not exaggerate the effects of mTBI on acute neurological dysfunction, assessed by duration of LORR. It may correlate with that the injury are too mild to cause accumulated effects on the acute neurological test. In addition, the adaptive responses may protect them from exaggerated injuries.

Memory decline is a most common complaint in mTBI patients, even up to 1 year after TBI^19, 21^. However, most studies on mTBI focused on short-term pathological, pathophysiological and behavioral changes, in the range of hours to 10 days post-mTBI^16–18^. In the current study, the memory function was assessed at 2, 8 and 12 weeks after the last injury by Morris water maze to determine both short-term and long-term effects of rmTBI on memory function. We found that rmTBI mice showed significant memory deficits compared with control mice from 2 to 12 weeks after the last injury. It indicates that rmTBI does have a chronic effect on memory impairments in addition to short-term memory deficits. This is the first study to examine the chronic effect of rmTBI on memory functions in a mouse model, which not only provides valuable in formation of chronic effects of rmTBI on memory functions but also suggests that long-term monitoring and interventions are imperative to recover the memory function in rmTBI patients. Moreover, the data of probe tests that the passing times in rmTBI mice were 12.5% of controls at 2 weeks, 42% of controls at 8 weeks and 52% of controls at 12 weeks, showed that the memory impairment was gradually recovered, which highly indicated that early intervention and treatment may be beneficial for rmTBI individuals to accelerate the improvement of memory functions.

To investigate the molecular mechanism of rmTBI-induced memory deficits, we performed gene expression profiling in rmTBI mice and control mice. The most dramatic memory deficit was observed at 2 weeks after the last injury, and the hippocampus, vulnerable to injury even with mild brain trauma, plays a critical role in learning and memory. We specifically profiled the whole genome gene expression in the hippocampus one day after the behavioral tests at 2-week time point. 87 differentially expressed genes in rmTBI mice were classified into transcription factor, transporter, receptor and membrane traffic protein etc. categories, which belonged to apoptotic process, response to stimulus, metabolic process, transcription regulation and enzyme regulator etc. functional groups. In addition, the dysregulated genes were also involved in synaptic vesicle trafficking, ionotropic glutamate receptor pathway, Huntington disease and Parkinson disease pathways. It is worth to note that majority of these altered genes were involved in pathological processes which promote development and progression of neurodegenerative diseases.

Neuroinflammation has been suggested to exaggerate the outcomes of neurodegenerative diseases. Several studies revealed that traumatic CNS injury could trigger severe systemic effects that lead to inflammation and pathological autoimmunity^27, 28^. Consistent with previous reports, we found rmTBI triggered upregulations of genes that were involved in pro-inflammatory response. Lcp1 (encoding L-plastin)was suggested to regulate the function of integrin in leukocytes^29^, which is important for leukocytes infiltration to the CNS^30^ and in turn induction of neuroinflammation. Another up-regulated gene is Neu1, which has been found to regulate Toll-like receptor (TLR) activation^31^. TLRs play an important role in the pathophysiology of infectious diseases, inflammatory diseases, and possibly in autoimmune diseases.Errfi1 (also known as Mig6) was another dysregulated gene, which is an immediate early gene transcriptionally induced by a divergent array of extracellular stimuli and has a possible role in the response to persistent stress^32^. In general, the data set demonstrated a predominantly inflammatory response, as the majority of pro-inflammatory genes were up-regulated and anti-inflammatory genes were down-regulated.

Free radicals produced under oxidative stress attack macromolecules, especially DNA, and in turn induce apoptosis. Within the differentially expressed genes, genes promoting oxidative stress were upregulated in the hippocampus from the rmTBI mice. Cyb5r1 is believed to be associated with the oxidative state of cells, and Erp29, is involved in the processing of secretory proteins within the endoplasmic reticulum (ER) and has been shown to take part in the ER stress signaling^33^. Therefore, it is possible that rmTBI could trigger oxidative stress responses in the hippocampus, leading to consequent neural dysfunction.

The ubiquitin proteasome system (UPS) controls the turnover of innumerable cellular proteins. It targets misfolded or unwanted proteins for general proteolytic destruction and tightly controls destruction of proteins involved in development and differentiation, cell cycle progression, apoptosis and many other biological processes. Dysregulation of the UPS is believed to be both a cause and result of neurodegenerative diseases^34–38^. In the rmTBI mice, two genes involving E3 protein-ubiquitin ligase were dysregulated. These are Fbxo10, as a component of the SCF (SKP1-CUL1-F-box protein)complex which acts as an E3 protein-ubiquitin ligase^39^, and Stub1 *per se* as an E3 ubiquitin-protein ligase. Studies revealed that Stub1 was upregulated in the hippocampus of the SAMP8 mice, which is a typical animal model to investigate the fundamental mechanisms of age-related learning and memory deficits associated with neuronal degeneration^40, 41^. It has been shown that down-regulation of Stub 1 after treatment with a Chinese medicine could ameliorate age-related learning and memory deficits^40, 41^. These results indicatedthat protein degradation and quality control is one of rmTBI’s effects on the brain.

Taken together, our results indicated that rmTBI may facilitate neuronal apoptosis. The data also provides insights into the possible mechanisms of rmTBI contributing to memory impairments and increased risk of neurodegenerative diseases^1–3^. Future studies are warranted to further examine pathophysiological changes and molecular alterations with proteomics-based analysis after rmTBI at different time points and to find novel valid targets for the development of effective rmTBI therapies.

## Materials and Methods

### rmTBI mouse model

Animal experiment protocols were approved by the University of British Columbia Animal Care and Use Committee, and the experimental procedures were carried out in accordance with the guidelines and regulations of The University of British Columbia Animal Care and Use Committee and Biosafety Committee. Male C57BL/6 mice of 10-week old were housed in the animal facility under standard conditions, 22 ± 1 °C with a 12:12 hr light- dark cycle. After 2 weeks housing for adapting to the laboratory environment, 17 micereceived sham treatment and 17 mice received rmTBI treatment. The modified weight drop device consists of a hollow Plexiglas tube with 2 cm in diameter and 40 cm length, a cylindrical-shaped acrylic stick (20 g) and a foam platform. The tube is kept vertical to the surface of the mouse head and guides the freely falling acrylic stick onto the head. The diameter of the upper part of the stick is 1.8 cm, which allows little lateralization of the stick to hit the head. The end of the stick is flat, round and 1 cm in diameter which will hit the top of the mouse head encompassing the area over the frontal and parietal bones. The mice were anesthetized by 2.5% is oflurane and the disappearance of extremity reflex stimulated by the rear foot toe pinch was considered to be suitable for injury. The mouse was quickly placed in a prone position on the platform so that the mouse head was closely underneath the lower opening of the Plexiglas tube. Next, the stick was released from 25 cm height. After consciousness (indicated by return of righting reflex and mobility) was regained, the mouse was put back to its home cage. The control mouse was anesthetized and placed on the platform in the same fashion as the injured ones, but without any injury. rmTBI mice received the concussive-like head injury once a day from Monday to Friday for 5 uninterrupted weeks, while control mice were treated following the same procedure except for concussive-like head injury. Behavioral tests were performed on mice in control group and rmTBI group at 2, 8 or 12 weeks after sham treatment or rmTBI treatment, respectively. The time line of the whole experiment is shown in Fig. 1A. The mice were sacrificed after water maze test at each time point.

### Acute neurological evaluation

Acute neurological evaluation was performed every day right after the injury was delivered by recording the time of loss of consciousness (LOC). LOC was evaluated by the duration of the loss of righting reflex (LORR). LORR was measured as the time interval between loss of the righting reflex and regain of the righting reflex^42^. Convulsion was also recorded throughout the acute evaluation period. Briefly, the mouse was placed in a clear Perspex observation box right after the injury was delivered. While recording for the time of LORR, the number and intensity of convulsion were also recorded. The duration of each convulsion was graded as short (1 to 10 s), medium (11 to 30 s), or long (≥31 s). According to the Charles River Laboratories grading system, the intensity of each convulsion was classified into mild, moderate, and severe convulsion^43^. Visible foreleg paralysis was recorded if the forelegs were paralyzed visibly following the injury for more than 1 hour.

### Wire hanging test

In order to measure motor neuromuscular activities and motor coordination, the endurance of wire hanging testwas measured by placing the mouse on top of a wire mesh (1 × 1 cm grid) which was taped around the edge and suspended 50 cm above a soft bedding material. The mesh was gently shaken so that the mouse griped the wire and then it was turned upside down and the amount of time spent holding on to the mesh with all the four legs was recorded, up to a maximum of 60 seconds.

### Morris water maze

The Morris water maze test was performed as we previously described^44, 45^. Briefly, a 1.5-m-diam, water-filled, cylindrical tank was used to perform Morris water maze. Extra-maze visual cues with different colors and shapes for orientation were permanently placed on the four walls around the tank. The temperature of the water in the tank was kept constant at 22 ± 1 °C. A 10-cm-diam platform was placed in a certain quadrant of the tank. The procedure consisted of 1 day of visible platform tests and 4 days of hidden platform tests, plus a probe trial 24 h after the last hidden platform test. In the visible platform test, the platform was lifted 0.5 cm above the water surface in the southeast, northeast, northwest, southwest quadrant and the center of the pool respectively in each trial. There were 5 contiguous trials, with an inter-trial interval of 1 hour. Mice were placed next to and facing the wall of the tank successively in north (N), east (E), south (S), and west (W) positions. In each trial the mouse was allowed to swim until it found the platform, or until 60 seconds had elapsed. In the latter case, the mouse was guided to the platform where it remained for 20 seconds before being returned to the cage. In the hidden platform tests, the platform was placed 0.5 cm below the water surface in the southeast quadrant and mice were trained for 5 trials per day from all the N, E, S, W positions with an intertrial interval of 1 hour. The probe trial was conducted by removing the platform and placing the mouse next to and facing the N side. The time spent in the previously platform quadrant (southeast quadrant) was measured in a single 60 seconds trial. Tracking of animal movement was achieved with ANY-maze video-tracking system.

### RNA preparation

2 weeks after the last injury, behavioral tests were performed in rmTBI mice and control mice. One day after all the behavioral tests, RNA was extracted from the hippocampi of 3 mice in control group and 3 mice in rmTBI group, respectively. Briefly, the mice were decapitated and the isolated hippocampi were stored immediately at −80 °C. Total RNA was extracted from hippocampi using TRI Reagent (Sigma-Aldrich, Inc., St. Louis, MO).

### Illumina whole genome gene expression assay

The complementary RNA (cRNA) was amplified from an input of 500 ng total RNA using the Illumina TotalPrep RNA Amplification Kit (Applied Biosystems Inc., Foster City, CA, AMIL1791) following the manufacturer’s instruction. The cRNA samples were then assessed for quality by measuring A260/A280. All of them should fall in the range of 1.7 to 2.1. The Illumina’s MouseWG-6 v 2.0 Expression BeadChip containing more than 45, 200 well annotated RefSeq transcripts allowed 6 samples to be interrogated in parallel on a single Bead chip. Labeled and amplified cRNAs (1.5 μg/array) were hybridized to Illumina’s Sentrix® Mouse-6 Expression Bead chips at 58 °C for 16 h according to Illumina® Whole-Genome Gene Expression with IntelliHyb Seal System Manual. The array was washed and stained with 1 μg/ml cyanine3-streptavidin then scanned using an Illumina BeadStation 500 G – Bead Array Reader (Illumina, Inc., San Diego, CA). Reference, hybridization control, stringency and negative control genes were checked for proper chip detection. The results were extracted with the Illumina’s Bead Studio software with quantile normalization and background subtraction. The Illumina custom error model with multiple testing corrections (Benjamini & Hochberg false discovery rate) was applied to dataset to identify genes differentially expressed following injuries (filtered by Illumina’s “detection p-value < 0.05” and Diff. p-value < 0.05).

### Functional classification and pathway analysis

The altered genes with foldchange ≥1.2, p < 0.05, were analyzed by Panther 9.0 (http://www.pantherdb.org/).

### Statistics

The data of duration of LORR were analyzed by one-way ANOVA. The data of Morris water maze were analyzed by two-way ANOVA or two-tailed Student’s t-test. All data were presented as means ± SEM. P < 0.05 was considered as statistically significant.

## Electronic supplementary material

Supplemental information
