# Supplementary material for: Mild traumatic brain injury induces memory deficits with alteration of gene expression profile
Source: Sci Rep. 2017 Sep 7;7:10846. doi: 10.1038/s41598-017-11458-9 (PMC5589921; doi:10.1038/s41598-017-11458-9)
Supplement: Supplementary file 1 — Supplemental information [file 41598_2017_11458_MOESM1_ESM.doc]

**Mild traumatic brain injury induces memory deficits with alteration of gene expression profile**

Yawen Luo ^1,2^, Haiyan Zou ^1,2^, Yili Wu ^1,2^, Fang Cai ^1,2^, Si Zhang ^1^, and Weihong Song ^1,3^

^1^ Townsend Family Laboratories, Department of Psychiatry, The University of British Columbia, 2255 Wesbrook Mall, Vancouver, BC, V6T 1Z3, Canada.

^2^ These authors contributed equally to this work.

^3^ To whom correspondence should be addressed: Dr. Weihong Song, MD, PhD, FCAHS. Email:[weihong@mail.ubc.ca](mailto:weihong@mail.ubc.ca)

**Supplemental Table**

**Supplemental Table 1: Differentially expressed genes in rmTBI mice**

| **SYMBOL** | **PROBE_ID** | **ratio** | **P-value** |
| --- | --- | --- | --- |
| 1700009P03Rik | ILMN_2743487 | 0.643672 | 0.027873 |
| 2310061F22Rik | ILMN_1221168 | 1.232453 | 0.015675 |
| 2410018C17Rik | ILMN_1218311 | 1.215897 | 0.027843 |
| 2900057D21Rik | ILMN_2608309 | 0.780167 | 0.029212 |
| 4121402D02Rik | ILMN_1244356 | 0.747166 | 7.94E-06 |
| 4930438D12Rik | ILMN_2548916 | 0.658795 | 0.011112 |
| 5730406M06Rik | ILMN_1228707 | 0.655429 | 0.000899 |
| 5730589K01Rik | ILMN_2779636 | 1.291694 | 0.039674 |
| 5830411K21Rik | ILMN_1245709 | 0.64261 | 0.035695 |
| Atp6v0d1 | ILMN_2685365 | 1.184213 | 0.020743 |
| B3gat1 | ILMN_2708717 | 0.818275 | 0.015951 |
| Bat2 | ILMN_1254154 | 0.5791 | 0.037649 |
| Bzrap1 | ILMN_1229256 | 0.791519 | 0.000444 |
| Cables1 | ILMN_1217097 | 1.487305 | 0.000948 |
| Camkk2 | ILMN_1256263 | 0.693758 | 0.036714 |
| Ccdc85a | ILMN_2723052 | 0.668741 | 0.011695 |
| Ccnt1 | ILMN_2758087 | 0.774869 | 0.009915 |
| Cited2 | ILMN_2477221 | 1.238876 | 0.047342 |
| Cog8 | ILMN_2595383 | 1.403383 | 0.039754 |
| Cope | ILMN_2944646 | 1.324935 | 0.017463 |

**Supplemental** Table 1: Differentially expressed genes in rmTBI mice (continued)

| **SYMBOL** | **PROBE_ID** | **ratio** | **P-value** |
| --- | --- | --- | --- |
| Copz2 | ILMN_2647028 | 1.398398 | 0.00035 |
| Coq7 | ILMN_2729263 | 1.343595 | 0.013054 |
| Cpne2 | ILMN_2870487 | 1.578032 | 0.029799 |
| Cyb5r1 | ILMN_1243370 | 1.498097 | 0.038825 |
| D17Wsu92e | ILMN_1225137 | 0.769679 | 0.042815 |
| D4Wsu132e | ILMN_1236246 | 0.81496 | 0.008975 |
| D8Ertd738e | ILMN_2820559 | 1.158783 | 0.047822 |
| Dbndd2 | ILMN_1249905 | 1.181445 | 0.026006 |
| Dhx38 | ILMN_3161767 | 1.209764 | 0.008457 |
| Dlg4 | ILMN_2710764 | 0.758182 | 1.95E-09 |
| Eif3i | ILMN_2789601 | 1.285847 | 0.04231 |
| Erp29 | ILMN_1239185 | 1.236254 | 0.008949 |
| Errfi1 | ILMN_2714031 | 1.264025 | 1.72E-05 |
| Extl3 | ILMN_1239857 | 1.240578 | 0.000343 |
| F730003H07Rik | ILMN_1259069 | 0.731846 | 0.012623 |
| Fbxo10 | ILMN_2529395 | 1.207003 | 0.01693 |
| Fibp | ILMN_1260261 | 1.180264 | 0.045779 |
| Gdi1 | ILMN_2632299 | 0.824895 | 0.042815 |
| Gdi1 | ILMN_2630975 | 0.768919 | 0.012682 |
| Gpx4 | ILMN_2684855 | 1.1991 | 0.00069 |

**Supplemental Table 1: Differentially expressed genes in rmTBI mice (continued)**

| **SYMBOL** | **PROBE_ID** | **ratio** | **P-value** |
| --- | --- | --- | --- |
| Gria2 | ILMN_3122922 | 0.828443 | 0.007989 |
| Gstz1 | ILMN_1229964 | 1.284163 | 0.024921 |
| Gtf2h3 | ILMN_1243127 | 1.243327 | 0.037354 |
| Hmg20a | ILMN_2968479 | 0.796552 | 6.55E-05 |
| Hnrpl | ILMN_2627690 | 0.756669 | 0.009404 |
| Kcna2 | ILMN_2727857 | 0.737461 | 7.94E-06 |
| Kcnma1 | ILMN_2723799 | 0.687341 | 0.000899 |
| Lbh | ILMN_2816180 | 0.828177 | 0.042815 |
| Lbh | ILMN_1233545 | 0.597619 | 0.003483 |
| LOC100044948 | ILMN_1244853 | 1.189998 | 0.029748 |
| LOC100048645 | ILMN_2635167 | 1.243729 | 0.045779 |
| LOC383942 | ILMN_2536518 | 0.756524 | 0.045779 |
| LOC433749 | ILMN_1219914 | 1.205532 | 0.047822 |
| Lrpprc | ILMN_2683718 | 0.680503 | 0.047342 |
| Mapk8ip1 | ILMN_2871628 | 1.258849 | 0.00035 |
| Mrpl38 | ILMN_2664049 | 1.276814 | 0.008949 |
| Mtap6 | ILMN_2439378 | 0.635724 | 0.007989 |
| mtDNA_ATP6 | ILMN_2470277 | 0.851151 | 3.33E-33 |
| Myst2 | ILMN_2665161 | 0.751351 | 1.95E-09 |
| Nedd4b | ILMN_1243947 | 0.733857 | 0.010583 |

**Supplemental Table 1: Differentially expressed genes in rmTBI mice (continued)**

| **SYMBOL** | **PROBE_ID** | **ratio** | **P-value** |
| --- | --- | --- | --- |
| Nedd4b | ILMN_1243947 | 0.733857 | 0.010583 |
| Neu1 | ILMN_2708906 | 1.210429 | 0.001948 |
| Nt5c | ILMN_1223097 | 1.23348 | 0.034 |
| Pcdh17 | ILMN_2804444 | 0.864059 | 0.015157 |
| Plec1 | ILMN_2710419 | 1.191097 | 0.045779 |
| Polr1c | ILMN_1252845 | 1.307677 | 0.00035 |
| Prickle1 | ILMN_1224069 | 0.748083 | 0.039885 |
| Rab26 | ILMN_2718406 | 1.200096 | 0.028952 |
| Rasl11a | ILMN_2932662 | 1.876731 | 0.006037 |
| Rnasen | ILMN_3144358 | 0.749248 | 0.038825 |
| Rph3a | ILMN_2595600 | 0.736737 | 0.018546 |
| Rreb1 | ILMN_1255511 | 0.773705 | 0.014142 |
| scl0002975.1_346 | ILMN_2448404 | 0.603119 | 2.06E-25 |
| Scn2a1 | ILMN_1214686 | 0.809605 | 0.034 |
| Sh2b1 | ILMN_1256412 | 1.275731 | 0.013073 |
| Sidt2 | ILMN_1258738 | 0.772042 | 0.002868 |
| Slc25a38 | ILMN_2946905 | 1.27076 | 0.003285 |
| Ssr4 | ILMN_1255237 | 1.216832 | 0.020459 |
| Stub1 | ILMN_2628532 | 1.405201 | 0.008986 |
| Sult1a1 | ILMN_2745370 | 1.570713 | 0.00069 |
| Surf4 | ILMN_2595846 | 1.292928 | 0.006445 |
|  |  |  |  |

**Supplemental Table 1: Differentially expressed genes in rmTBI mice (continued)**

| **SYMBOL** | **PROBE_ID** | **ratio** | **P-value** |
| --- | --- | --- | --- |
| Syn1 | ILMN_1235133 | 0.841076 | 0.00035 |
| Syne1 | ILMN_1248614 | 0.687617 | 0.034 |
| Tmem50b | ILMN_2671286 | 0.854337 | 0.022677 |
| Tnfrsf21 | ILMN_2464573 | 0.670815 | 0.010622 |
| Tsta3 | ILMN_1221145 | 1.228067 | 0.002175 |
| Ttc33 | ILMN_2713039 | 0.845698 | 0.012982 |
| Zdhhc4 | ILMN_1239603 | 1.322828 | 3.59E-05 |
| Zranb1 | ILMN_1249755 | 0.820862 | 0.00377 |
| Zswim6 | ILMN_2524100 | 0.802577 | 0.00377 |
|  |  |  |  |
|  |  |  |  |
|  |  |  |  |
|  |  |  |  |
